# Risk loci for coronary artery calcification replicated at 9p21 and 6q24 in the Heinz Nixdorf Recall Study

**DOI:** 10.1186/1471-2350-14-23

**Published:** 2013-02-08

**Authors:** Sonali Pechlivanis, Thomas W Mühleisen, Stefan Möhlenkamp, Dirk Schadendorf, Raimund Erbel, Karl-Heinz Jöckel, Per Hoffmann, Markus M Nöthen, André Scherag, Susanne Moebus

**Affiliations:** 1 Institute for Medical Informatics, Biometry and Epidemiology, University Hospital Essen, Essen, Germany; 2 Institute of Human Genetics, University of Bonn, Bonn, Germany; 3 Department of Genomics, Life & Brain GmbH, University of Bonn, Bonn, Germany; 4 Clinic of Cardiology, West-German Heart Centre, University Hospital Essen, Essen, Germany; 5 Department of Dermatology, Venerology and Allergology, University Hospital Essen, Essen, Germany

**Keywords:** Coronary heart disease, Coronary artery calcification, Cohort study, Polymorphism, Metabochip

## Abstract

**Background:**

Atherosclerosis is the primary cause of coronary heart disease (CHD), preceding the onset of cardiovascular disease by decades in most cases. Here we examine the association between single nucleotide polymorphisms (SNPs) integrated on Metabochip and coronary artery calcification (CAC), a valid risk factor for CHD, in an unselected, population-based German cohort.

**Methods:**

The Metabochip is a custom iSELECT array containing >195,000 SNPs that was designed to support large-scale follow-up of putative associations for metabolic and cardiovascular-associated traits. We used generalized linear regression models to explore the impact of Metabochip SNPs on quantitative CAC in 4,329 participants.

**Results:**

The 9p21 variant, rs1537373, was most strongly associated (Beta = 0.30; 95% confidence interval (CI) = 0.21-0.39; p = 4.05x10^-11^) with quantitative CAC. The second strongest association with CAC was with rs9349379 in the phosphatase and actin regulator 1 gene, *PHACTR1*, (Beta = 0.30; 95% CI = 0.22-0.40; p = 4.67x10^-11^). Both SNPs remained nominally significant in dichotomized analyses for the presence of any CAC (odds ratio_rs1537373_ (OR) = 1.19; 95% CI = 1.07-1.31; p = 0.001 and OR_rs9349379_ = 1.26; 95% CI = 1.14-1.40); p = 1.5x10^-5^). Fine mapping of the 9p21 and *PHACTR1* gene region revealed several other SNPs that were strongly associated with CAC.

**Conclusion:**

We demonstrate that SNPs near 9p21 and in *PHACTR1* that have previously been shown to be associated with CHD are strongly associated with CAC in the Heinz Nixdorf Recall Study cohort. Our findings suggest that the 9p21 and 6q24 loci might be involved in cardiac outcome via promoting development of atherosclerosis in the coronary arteries.

## Background

Atherosclerosis is the primary cause of coronary heart disease (CHD), one of the key causes for death and morbidity in the industrialized world [1,2]. Atherosclerosis in the arterial wall precedes the onset of most cases of clinically apparent cardiovascular disease by decades [3]. Coronary artery calcification (CAC) is one of the most sensitive and specific markers of coronary atherosclerotic plaque burden. CAC quantification has been shown to improve the ability to predict future CHD events [4,5]. Numerous environmental and genetic factors have been demonstrated to influence CAC, and strong heritability of CAC has been shown to account for around 40% of the observed genetic variance [6-8]. Several CHD risk factors, including male gender, age, elevated blood pressure, low-density lipoprotein and diabetes, have been shown to be related with CAC [9-12].

Several genome-wide association studies (GWASs) identified novel gene loci for CHD, to which CAC is one of the precursors [13-25]. The CHARGE consortium recently reported strong associations between CAC and several single nucleotide polymorphisms (SNPs) located on chromosome 9p21 and the phosphatase and actin regulator 1 gene, *PHACTR1*[26].

While genome-wide findings have often been replicated for individual SNPs (at α = 5 × 10^-8^), causal variants could not be identified using classical fine mapping of a limited number of markers in most cases due to inadequate sample availability or the lack of defined phenotypes information. The next generation of high-density custom SNP array, the Metabochip, was designed to support fine-mapping. About 200,000 SNPs related to atherosclerotic-cardiovascular and metabolic traits are embedded on the Metabochip [27]. Thus, the Metabochip is a state-of-the-art summary of relevant genetic variability which will be explored in this study.

Only few studies to date have assessed the role of genetic factors for subphenotypes, such as CAC, of coronary artery disease (CAD). The examination of specific genetic variations affecting CAC may help explain the essential mechanisms leading to CHD and might aid development of strategies to predict and prevent CHD. Here we aimed to further this effort by evaluating SNPs associated with CAC using the Metabochip in unselected, population-based participants in the German Heinz Nixdorf Recall Study. Since the Metabochip also includes the loci detected by the CHARGE consortium [26], our study can also be regarded as an independent replication in a second cohort.

## Methods

### Study population

We used baseline data from the Heinz Nixdorf Recall Study (Risk Factors, Evaluation of Coronary Calcium and Lifestyle), comprising 4,814 participants spanning 45–75 years of age. Participants were randomly selected from registration lists between 2000 and 2003 from the densely populated Ruhr metropolitan area in Germany (residents of Essen, Bochum and Mühlheim). We followed a study which previously described study rationale and design [28]. Information for genotype, gender, age and CAC was available for 4,329 of 4,814 participants (Table 1). Baseline CHD was assessed as self-report of previous events (myocardial infarction and/or revascularization of coronary arteries including balloon dilation and coronary bypass surgery). CAC was assessed by non-enhanced electron-beam CT (C-150 scanner, GE Imatron, San Francisco, CA, USA), as previously described [28]. The study was approved by the local ethics committees, and conducted in accordance with the Declaration of Helsinki and guidelines for good epidemiological practice, including extended quality management procedures and recertification according to DIN ISO 9001:2001. Informed consent was obtained from all participants.

**Table 1:** Characteristics of the study population*

**Variable**	**Males (n = 2158)**	**Females (n = 2171)**
Age, years^†^	59.8 ± 7.80	59. 7 ± 7.80
Known coronary heart disease*	223 (10.3)	62 (2.9)
CAC score ^‡^	76. 5 (6.7; 351.8)	2.1 (0.0; 43.8)
Log_e_(CAC score + 1)^‡^	4.35 (2.0; 5.9)	1.13 (0.0; 3.8)
Presence of any CAC*		
0	337 (15.6)	948 (43.7)
> 0	1821 (84.2)	1223 (56.3)

† Data are given as mean ± SD.

* Data are given as number (percentage).

^‡^ Data are given as median (quartile 1; quartile 3).

### SNP genotyping

Lymphocyte DNA was isolated from EDTA anti-coagulated venous blood using the Chemagic Magnetic Separation Module I (Chemagen, Baesweiler, Germany). Genotyping was performed on Metabochip BeadArrays (Illumina, San Diego, CA, USA). Quality control utilized the PLINK (v1.07) software package [29]. Genotyping was conducted on a total of 4,570 samples, of which 52 samples were excluded from data analysis because of i) gender inconsistency, ii) identical-by-state was estimated to measure the degree of recent shared ancestry for a pair of individuals. We removed one individual from each pair of putatively-related samples defined as an identity by descent value greater than 0.185 or iii) a sample call rate less than 97%. Out of the 196,725 SNP probes arrayed on Metabochip, 4,464 were excluded because they had call rates less than 95% or because the Hardy Weinberg equilibrium P_exact_ was less than 1×10^-4^.

### Statistical analyses

The association between each SNP and the log_e_ transformed CAC score +1 was analysed using linear regression with adjustment for age and gender in an additive genetic model as described previously [30]. Logistic regression was used for the sensitivity analysis of binary CAC outcome (CAC > 0 as presence and CAC = 0 as absence of CAC adjusted for gender and age). All analyses were repeated after excluding individuals who had CHD at baseline (n = 285), resulting in a study sample size of 4,035. Gender-stratified analyses were also performed for the top SNPs. A large proportion of Metabochip consists of SNPs for the fine mapping of loci that were robustly associated in meta-analysis [27]. To address the between-marker linkage disequilibrium (LD), we first used the pruning technique implemented as “clump” procedure in PLINK [29]. This procedure selects all SNPs having nominal p-values ≤ 0.0001 that have not already been clumped (denoted as index SNPs). Each index SNP then forms clumps with other SNPs that are within ± 250 kb from the index SNP and that are in LD with the index SNP (r^2^ ≥ 0.50). If separate signals from the same locus were associated with CAC, a conditional analysis was performed in PLINK using linear regression adjusted for the covariates age and gender, and the lead SNP at the neighboring signals. We applied a conservative Bonferroni correction with a significance level of α = 2.6×10^-7^ (0.05/192261). A p-value between 2.6×10^-7^ and 1×10^-5^ was considered as moderate evidence for association. SNPs having p ≤ 1.0×10^-5^ within the "clump" of significant SNPs are reported in Additional file 1: Table S1. All other SNPs with p ≤ 1.0×10^-5^ are reported in Additional file 2: Table S2. SNPs reported to be associated with CAC by the CHARGE consortium and SNPs reported to be associated with CHD by the CARDIOGRAM consortium are listed in Additional file 3: Table S3 [23,26]. Power calculations were made using the QUANTO Version 1.2.3 software (http://hydra.usc.edu/gxe) considering a minor allele frequency  ≥ 20% and α = 2.6×10^-7^ (two-sided). Power was over 90% for the 3,044 individuals with CAC > 0 assuming a standard normally distributed quantitative trait locus and a standardized effect size of 0.2 in units of standard deviations for each risk allele under an additive mode of inheritance without dominance effects. Thus, our study was well powered to detect a relatively strong effect size of quantitative CAC predisposing variants when controlling for multiple testing. For the gender-stratified analyses with CAC > 0, power was over 38% for males (n = 1,821) and over 12% for females (n = 1,223). We reported 95% confidence intervals (95% CI) for all estimates and nominal, two-sided p-values that are, unless otherwise stated, not adjusted for multiple testing. All analyses were done using PLINK v1.07 [29].

## Results

### SNPs associated with coronary artery calcification

The lead SNPs having p ≤ 2.6×10^-7^ in each clump was identified by calculating the effect size estimators (Betas) for the linear regression models with quantitative transformed CAC (log_e_(CAC score + 1)) (Table 2). The strongest association was observed for rs1537373 on chromosome 9p21 (Beta = 0.30; 95% CI = 0.21-0.39; p = 4.05×10^-11^). Even in the dichotomized CAC analysis, rs1537373 remained nominally significant (p = 0.001) with an increased risk for the presence of CAC (odds ratio for each G risk allele = 1.19; 95% CI = 1.07-1.31). The rs10965219 SNP was also associated with quantitative CAC (Beta = 0.25; 95% CI = 0.16-0.39; p = 1.32×10^-7^). It belongs to a separate clump and is located 50kb upstream of rs1537373. In the clump including rs1537373, 45 additional SNPs are located that were also associated with CAC (p ≤ 1×10^-5^, Additional file 1: Table S1). Similarly, 66 SNPs were also associated (p ≤ 1.0×10^-5^) with CAC within the clump including the rs10965219 SNP. Apart from SNPs at the chromosome 9p21 locus, the rs9349379 SNP located in the phosphatase and actin regulator 1 gene (*PHACTR1*) on chromosome 6p24 was significantly associated with CAC (Beta = 0.30; 95% CI = 0.22-0.40; p = 4.67×10^-11^). No other SNP besides rs9349379 was present in this clump that was significantly associated with quantitative CAC (p ≤ 1.0×10^-05^). SNP rs9349379 was also nominally significant in the dichotomized analysis with an increased risk for presence of CAC (odds ratio for each G risk allele = 1.26; 95% CI = 1.14-1.4; p = 1.47×10^-5^). In a sensitivity analysis, we excluded all participants with prevalent CHD at baseline. The beta estimates for this analysis hardly changed (Table 2), with the largest change (Beta = 0.04, from 0.30-0.26) observed for rs1537373 at chromosome 9p21. In an explorative gender-stratified analysis (Table 3), the chromosome 9p21 SNPs showed a descriptively stronger effect for association with CAC in males compared to females. On the other hand, the effect of association for rs9349379 in the *PHACTR1* gene with CAC was stronger in females as compared to males. However, only rs10965219 showed a marginally significant genotype and gender interaction (after Bonferroni correction) (Beta_rs10965219xgender_ = −0.23; 95% CI = −0.4 to −0.05); p = 0.01).

**Table 2:** **Results of the genetic association analyses for log**_**e**_**(CAC score + 1) using Metabochip SNPs (additive genetic model), gender and age as predictors in a linear regression model**

**CHR**	**Gene**	**SNP**	**Physical position**	**Minor allele (MAF)**	**log**_**e**_**(CAC score + 1)**		**log**_**e**_**(CAC +1) without CHD**		**dichotomized CAC**	
					**Beta (95% CI)**	**P**	**Beta (95% CI)**	**P**	**OR (95% CI)**	**P**
9	*CDKN2A/2B*	rs1537373	22093341	G (0.47)	0.30 (0.21-0.39)	4.05x10^-11^	0.26 (0.17-0.35)	1.52x10^-8^	1.19 (1.07-1.31)	0.001
		rs10965219	22043687	G (0.48)	0.25 (0.16-0.39)	1.32x10^-7^	0.23 (0.14-0.32)	1.28x10^-6^	1.16 (1.05-1.29)	0.005
6	*PHACTR1*	rs9349379	13011943	G (0.39)	0.30 (0.22-0.40)	4.67x10^-11^	0.31 (0.22-0.40)	3.48x10^-11^	1.26 (1.14-1.4)	1.47x10^-5^

*CHR *Chromosome number; *SNP *single nucleotide polymorphism; *MAF* minor allele frequency from our data; *95% CI *95% confidence interval; *CAC *coronary artery calcium and *CHD *coronary heart disease.

**Table 3:** **Results of the genetic association analyses for log**_**e**_**(CAC score + 1) using Metabochip SNPs (additive genetic model) and age as predictors in a linear regression model for females and males**

**CHR**	**Gene**	**SNP**	**Physical position**	**Gender-SNP Interaction**	**Females**		**Males**		
				**Beta (95% CI)**	**P**	**Beta (95% CI)**	**P**	**Beta (95% CI)**	**P**
9	*CDKN2A/2B*	rs1537373	22093341	−0.07 (−0.25 to 0.11)	0.44	0.27 (0.15-0.39)	1.28x10^-5^	0.34 (0.21-0.47)	6.76x10^-7^
		rs10965219	22043687	−0.23 (−0.41 to -0.06)	0.01	0.13 (0.01-0.26)	0.03	0.36 (0.23-0.50)	1.25x10^-7^
6	*PHACTR1*	rs9349379	13011943	0.08 (−0.01 to 0.27)	0.37	0.35 (0.23-0.48)	3.5x10^-8^	0.27 (0.14-0.41)	7.58x10^-5^

*CHR *Chromosome number; *SNP *single nucleotide polymorphism; *MAF* minor allele frequency from our data; *95% CI *95% confidence interval; *CAC *coronary artery calcium.

Since several separate signals at the 9p21 locus were associated with CAC in our study, we carried out conditional analyses to better understand these associations. The effect of rs1537373 remained (Beta = 0.24; 95% CI = 0.13-0.35; p = 1.64×10^-5^), even after adjusting for the neighbouring lead SNP rs10965219. However, effect of rs10965219 almost disappeared (Beta = 0.06; 95% CI = −0.002 to 0.22); p = 0.06) after adjusting for rs1537373. Of the 2 associated 9p21 region SNPs only rs1537373 remained significantly associated with CAC in the conditional analysis suggesting that rs1537373 is the only independent 9p21 region signal to be associated with CAC in our study.

### *In silico* analyses of previously reported loci for CAC (CHARGE) and CAD (CARDIOGRAM) for the association with CAC using Metabochip

We also looked to verify associations of SNPs reported by the CHARGE consortium with CAC in our independent cohort [26]. Out of the 8 SNPs reported by CHARGE, 4 were present on the Metabochip. We confirmed the association of rs1333049 (near *CDKN2A/B*), rs9349379 (*PHACTR1)* and rs2026458 (*PHACTR1*) with CAC in our study with same direction of effect (all p < 2.84×10^-5^; Additional file 1: Tables S1 and Additional file 2: Table S2). We observed no evidence for an association of SNPs in the *COL4A1/COL4A2* gene (rs3809346 and rs4773144) with CAC in our study cohort (minimal uncorrected p = 0.19). We also looked for association of other SNPs located in this region that were present on the Metabochip with CAC. This included a total of 1,379 SNPs, of which 35 had p-values between 0.005–0.05 (data not shown). Other CHARGE discovery SNPs rs6604023 (near *CDC7*), rs6783981 (*SERPINI1*), rs17676451 (*HAL*) and rs8001186 (near *IRS2*) were not present on Metabochip. As a proxy for these, we also looked for SNPs present in the *SERPINI1* and *HAL* gene regions and located ± 100 kb from rs6604023 and rs8001186, respectively. No SNP located within ±100 kb of rs6604023, rs8001186 or rs6783981 was associated with CAC (all p > 0.09), and only the rs1085997 SNP, located 9.5 kb downstream of rs17676451 (r^2^ = 0.01, D' = 0.57), was found to be nominally associated with CAC (Beta = 0.19; 95% CI = 0.05-0.32; p = 0.006).

Similarly, we assessed the association of the CAD SNPs reported by the CARDIOGRAM consortium with the occurrence of CAC in our study [23]. Nineteen of 25 SNPs reported by the CARDIOGRAM consortium were present on the Metabochip. Nine SNPs were nominally associated with CAC in our study (all p < 0.04). Seven SNPs [rs599839 (*SORT1*), rs17465637 (*MIA3*), rs12526453 (*PHACTR1*), rs1746048 (*CXCL12*), rs9982601 (*MRPS6*), rs17114036 (*PPAP2B*) and rs3825807 (*ADAMTS7*)] showed same direction of effect for CAC and rs2306374 (*MRAS*) and rs579459 (*ABO*) SNPs showed opposite direction of effect for CAC as reported by CHARGE CAC GWAS (Additional file 3: Table 3).

## Discussion

Here we identified several SNPs at two loci on chromosome 9p21 and on chromosome 6p24 (*PHACTR1*) that are strongly associated with CAC in a population-based cohort. In addition, we confirm associations of rs1333049 (near *CDKN2A/2B*) and rs9349379 (*PHACTR1*) with CAC that were previously reported by the CHARGE consortium in an independent cohort.

Recent GWASs have identified a number of SNPs at 9p21 that are strongly associated with CAC and CHD [18,19,21,22,26,31]. A study to identify potential transcriptional regulatory elements at the 9p21 CAD locus showed that rs10757278, which has been consistently associated with CHD, lies in the 9^th^ enhancer region, where most of the CAD associated variants are located, and disrupts a transcription binding site for the signal transducer and activator of transcription 1 (STAT1) [32]. STAT1 mediates response to inflammation, which is associated with angiogenesis and atherosclerotic pathogenesis in endothelial tissue [32-34]. The rs10757278 SNP is in strong LD with the variant detected here, rs1537373 (r^2^ = 0.93, D' = 0.97), and is also strongly associated with quantitative CAC by itself (Additional file 1: Table S1). Recent studies showed that the CAD risk interval regulates cardiac CDKN2A/B expression, and affects CAD progression by altering vascular cell proliferation [35,36]. Thus, using the Metabochip fine-mapping approach, we demonstrate that several common variants lying in the important region on chromosome 9p21 are associated with CAC. Our independent finding is a robust replication of the CAC GWAS finding of the CHARGE consortium. Taken together, the chromosome 9p21 region is the most interesting candidate for advanced functional studies or animal models focussing on atherosclerosis.

The second strongest signal was found for rs9349379 in the *PHACTR1* gene. This SNP lies in the intronic region of *PHACTR1* gene. The LD values between rs9349379 and two previously associated CHD SNPs, rs12526453 and rs1332844, are r^2^ = 0.32, D' = 0.96 and r^2^ = 0.37, D' = 1, respectively [14,20,23]. PHACTR1 is an inhibitor of protein phosphatase 1 (PP1), an enzyme that dephosphorylates serine and threonine residues in a range of proteins [37]. PHACTR1 contains 8 predicted sites for protein kinase A phosphorylation and 7 predicted sites for protein kinase C phosphorylation, and phosphorylation at these sites is suggested to regulate PP1-substrate interactions [37]. Although both loci were strongly associated with CAC in our study, we were unable to elucidate the cause behind these associations. Additional functional studies, combined with SNP data, are needed to discover the cause behind SNP associations.

Our top findings varied somewhat for gender. Of course, such findings require a replication in larger samples. On the other hand, gender-specific effects might be more frequently detectable for CAC given the very different CAC distributions in men and women [38]. Finally, the absence of previously reported association of SNPs from CHARGE and CARDIOGRAM may be either due to the smaller numbers of participants investigated here, or may result from confounding factors that were not controlled for.

## Conclusion

We demonstrate that genetic variants near 9p21 and *PHACTR1* that have been previously shown to be associated with CHD are strongly associated with CAC in our study. These finding suggests that both loci might be involved in the cardiac outcome by promoting development of atherosclerosis in the coronary arteries.

## Competing interests

Stefan Möhlenkamp received <10KUD$ from AstraZeneca.

## Authors’ contributions

KHJ, RE, SM and SMö contributed to the conception, study design and data acquisition. SP conducted statistical analyses and contributed to the study design and interpretation of results. AS conducted statistical analyses and contributed to interpretation of results. DS acquired the data. MMN, TWM and PH carried out SNP genotyping. All authors contributed to manuscript preparation, and read and approved the final manuscript.

## Pre-publication history

The pre-publication history for this paper can be accessed here:

http://www.biomedcentral.com/1471-2350/14/23/prepub

## Supplementary Material

Additional file 1: Table S1 Result of the SNPs belonging to the same clump of the top associated SNP. The SNPs in the clump of the top associated SNP are selected based on pruning technique implemented as clump procedure in PLINK. SNPs marked in bold are the top associated SNP which are presented in Table 2. Click here for file

Additional file 2: Table S2 Result of the SNPs belonging to the same clump of SNPs associated with P ≤ 1x10^-05^ for log_e_(CAC score + 1). SNPs marked in bold are the top associated SNP of each clump. Click here for file

Additional file 3: Table S3 Result of association for log_e_(CAC score + 1) with SNPs associated with coronary artery disease in CARDIOGRAM and coronary artery calcification in CHARGE. Click here for file
