# Supplementary material for: Risk loci for coronary artery calcification replicated at 9p21 and 6q24 in the Heinz Nixdorf Recall Study
Source: BMC Med Genet. 2013 Feb 8;14:23. doi: 10.1186/1471-2350-14-23 (PMC3583714; doi:10.1186/1471-2350-14-23)
Supplement: Additional file 1: Table S1 — Result of the SNPs belonging to the same clump of the top associated SNP. The SNPs in the clump of the top associated SNP are selected based on pruning technique implemented as clump procedure in PLINK. SNPs marked in bold are the top associated SNP which are presented in Table 2. [file 1471-2350-14-23-S1.doc]

**Supplementary Table I:** Result of the SNPs belonging to the same clump of the top associated SNP. The SNPs in the clump of the top associated SNP are selected based on pruning technique implemented as clump procedure in PLINK. SNPs marked in bold are the top associated SNP which are presented in Table 2.

| CHR | SNP | Physical position | Minor allele (MAF) | BETA (95% CI) | P | HWE |
| --- | --- | --- | --- | --- | --- | --- |
| **9** | **rs1537373*** | **22093341** | **G (0.47)** | **0.30 (0.21;0.39)** | **4.05 x10^-11^** | **0.55** |
| 9 | chr9:22055002* | 22055002 | G (0.42) | 0.30 (0.20;0.39) | 2.40x10^-10^ | 0.81 |
| 9 | chr9:22057593* | 22057593 | G (0.42) | 0.30 (0.21;0.39) | 2.23x10^-10^ | 0.85 |
| 9 | chr9:22062638 | 22062638 | A (0.46) | 0.28 (0.19;0.37) | 6.19x10^-10^ | 0.24 |
| 9 | chr9:22062719 | 22062719 | G (0.42) | 0.29 (0.20;0.39) | 5.71x10^-10^ | 0.95 |
| 9 | chr9:22074310* | 22074310 | T (0.46) | 0.30 (0.21;0.39) | 6.91x10^-11^ | 0.55 |
| 9 | chr9:22078090 | 22078090 | T (0.46) | 0.28 (0.19;0.38) | 7.53x10^-10^ | 0.95 |
| 9 | chr9:22078094* | 22078094 | G (0.46) | 0.28 (0.20;0.38) | 7.48x10^-10^ | 1.00 |
| 9 | chr9:22088619* | 22088619 | G (0.46) | 0.29 (0.20;0.38) | 4.94x10^-10^ | 0.83 |
| 9 | chr9:22092165* | 22092165 | T (0.46) | 0.29 (0.20;0.38) | 4.28x10^-10^ | 0.74 |
| 9 | chr9:22093183* | 22093183 | T (0.42) | 0.28 (0.19;0.37) | 1.76x10^-09^ | 0.81 |
| 9 | chr9:22102241* | 22102241 | C (0.49) | 0.29 (0.20;0.38) | 2.55x10^-10^ | 0.72 |
| 9 | chr9:22102427 | 22102427 | G (0.49) | 0.29 (0.20;0.38) | 3.39x10^-10^ | 0.74 |
| 9 | chr9:22102599* | 22102599 | C (0.48) | 0.27 (0.18;0.36) | 3.24x10^-09^ | 0.98 |
| 9 | chr9:22105026* | 22105026 | G (0.49) | 0.29 (0.20;0.38) | 1.45x10^-10^ | 0.61 |
| 9 | chr9:22105286* | 22105286 | C (0.49) | 0.30 (0.21;0.39) | 1.17x10^-10^ | 0.63 |
| 9 | chr9:22106071* | 22106071 | C (0.48) | 0.28 (0.19;0.37) | 1.58x10^-09^ | 0.93 |
| 9 | chr9:22106220 | 22106220 | C (0.49) | 0.29 (0.20;0.38) | 1.45x10^-10^ | 0.61 |
| 9 | chr9:22109195* | 22109195 | C (0.49) | 0.22 (0.13;0.31) | 2.86x10^-06^ | 0.74 |
| 9 | chr9:22111349 | 22111349 | A (0.49) | -0.23 (-0.33;-0.14) | 3.57x10^-07^ | 0.91 |
| 9 | chr9:22114123 | 22114123 | A (0.47) | 0.28 (0.19;0.38) | 7.06x10^-10^ | 0.79 |
| 9 | chr9:22114140 | 22114140 | T (0.49) | 0.30 (0.21;0.39) | 1.26x10^-10^ | 0.83 |
| 9 | chr9:22114450 | 22114450 | G (0.46) | 0.27 (0.18;0.36) | 8.76x10^-09^ | 0.51 |
| 9 | rs10811656 | 22114472 | T (0.46) | 0.27 (0.18;0.36) | 9.46x10^-09^ | 0.49 |
| 9 | rs10757278* | 22114477 | G (0.46) | 0.27 (0.18;0.36) | 8.73x10^-09^ | 0.67 |
| 9 | chr9:22114630 | 22114630 | G (0.46) | 0.27 (0.18;0.36) | 8.73x10^-09^ | 0.49 |
| 9 | chr9:22115347* | 22115347 | C (0.47) | 0.29 (0.20;0.38) | 5.28x10^-10^ | 0.95 |
| 9 | rs1004638* | 22105589 | T (0.49) | 0.29 (0.20;0.38) | 1.90x10^-10^ | 0.61 |
| 9 | rs10116277* | 22071397 | T (0.46) | 0.29 (0.20;0.38) | 2.51x10^-10^ | 0.61 |
| 9 | rs10733376* | 22104469 | C (0.49) | 0.29 (0.20;0.38) | 3.37x10^-10^ | 0.57 |
| 9 | rs10738609* | 22104495 | G (0.47) | 0.26 (0.20;0.35) | 1.60x10^-08^ | 0.81 |
| 9 | rs10738610* | 22113766 | C (0.47) | 0.29 (0.20;0.38) | 5.34x10^-10^ | 0.93 |
| 9 | rs10757269* | 22062264 | G (0.47) | 0.29 (0.20;0.38) | 1.54x10^-10^ | 0.55 |
| 9 | rs10757274 | 22086055 | G (0.46) | 0.28 (0.19;0.37) | 7.96x10^-10^ | 0.83 |
| 9 | rs1333042 | 22093813 | G (0.48) | 0.30 (0.21;0.39) | 5.90x10^-11^ | 0.57 |
| 9 | rs1333043* | 22096731 | A (0.49) | 0.28 (0.19;0.37) | 7.22x10^-10^ | 0.53 |
| 9 | rs1333047* | 22114504 | T (0.47) | 0.28 (0.19;0.37) | 1.04x10^-09^ | 0.77 |
| 9 | rs1333049* | 22115503 | C (0.46) | 0.26 (0.17;0.36) | 1.36x10^-08^ | 0.61 |
| 9 | rs1412834* | 22100131 | C (0.49) | 0.28 (0.19;0.37) | 7.09x10^-10^ | 0.83 |
| 9 | rs1537371* | 22089568 | A (0.48) | 0.30 (0.21;0.39) | 5.15x10^-11^ | 0.63 |
| 9 | rs1537374* | 22106046 | G (0.49) | 0.29 (0.20;0.38) | 1.89x10^-10^ | 0.63 |
| 9 | rs1556516* | 22090176 | C (0.48) | 0.30 (0.21;0.39) | 4.82x10^-11^ | 0.57 |
| 9 | rs2383207* | 22105959 | G (0.49) | 0.29 (0.20;0.38) | 1.89x10^-10^ | 0.63 |
| 9 | rs4977575 | 22114744 | G (0.47) | 0.28 (0.19;0.39) | 8.15x10^-10^ | 0.88 |
| 9 | rs6475606* | 22071850 | T (0.46) | 0.29 (0.20;0.38) | 2.28x10^-10^ | 0.61 |
| 9 | rs9632884* | 22062301 | C (0.47) | 0.29 (0.20;0.38) | 2.07x10^-10^ | 0.49 |
|  |  |  |  |  |  |  |
| **9** | **rs10965219** | **22043687** | **G(0.48)** | **0.25 (0.16;0.39)** | **1.32 x10^-7^** | **0.31** |
| 9 | chr9:21985882 | 21985882 | T (0.49) | -0.21 (-0.30;-0.12) | 5.65x10^-06^ | 0.63 |
| 9 | chr9:21987872* | 21987872 | A (0.35) | 0.23 (0.14;0.33) | 1.65x10^-06^ | 0.62 |
| 9 | chr9:21988660 | 21988660 | A (0.44) | 0.21 (0.11;0.30) | 1.21x10^-05^ | 0.17 |
| 9 | chr9:21999698* | 21999698 | G (0.46) | -0.20 (-0.29;-0.11) | 1.23x10^-05^ | 0.59 |
| 9 | chr9:22000004* | 22000004 | A (0.35) | 0.25 (0.16;0.34) | 1.38x10^-07^ | 0.05 |
| 9 | chr9:22001642 | 22001642 | A (0.39) | -0.22 (-0.32;-0.13) | 2.37x10^-06^ | 0.95 |
| 9 | chr9:22003411 | 22003411 | T (0.45) | 0.23 (0.13;0.32) | 1.39x10^-06^ | 0.21 |
| 9 | chr9:22003805 | 22003805 | T (0.45) | 0.23 (0.14;0.32) | 1.26x10^-06^ | 0.19 |
| 9 | chr9:22005465 | 22005465 | A (0.39) | -0.22 (-0.32;-0.13) | 2.09x10^-06^ | 0.68 |
| 9 | chr9:22007101 | 22007101 | T (0.45) | 0.23 (0.14;0.32) | 7.41x10^-07^ | 0.20 |
| 9 | chr9:22007550 | 22007550 | G (0.45) | 0.23 (0.14;0.33) | 6.03x10^-07^ | 0.20 |
| 9 | chr9:22008781 | 22008781 | A (0.45) | 0.23 (0.14;0.32) | 7.58x10^-07^ | 0.14 |
| 9 | chr9:22009673* | 22009673 | C (0.45) | -0.21 (-0.30;-0.12) | 8.05x10^-06^ | 0.43 |
| 9 | chr9:22009732 | 22009732 | G (0.48) | 0.23 (0.14;0.32) | 4.75x10^-07^ | 0.70 |
| 9 | chr9:22013795 | 22013795 | A (0.47) | 0.24 (0.15;0.33) | 3.65x10^-07^ | 0.13 |
| 9 | chr9:22014351 | 22014351 | T (0.48) | -0.24 (-0.33;-0.15) | 2.77x10^-07^ | 0.79 |
| 9 | chr9:22014966 | 22014966 | G (0.45) | 0.24 (0.15;0.33) | 2.83x10^-07^ | 0.14 |
| 9 | chr9:22015240 | 22015240 | G (0.45) | 0.23 (0.14;0.32) | 1.16x10^-06^ | 0.09 |
| 9 | chr9:22016594 | 22016594 | A (0.42) | -0.22 (-0.31;-0.13) | 2.77x10^-06^ | 0.52 |
| 9 | chr9:22017402 | 22017402 | A (0.42) | -0.22 (-0.31;-0.13) | 2.55x10^-06^ | 0.60 |
| 9 | chr9:22018801 | 22018801 | G (0.47) | 0.24 (0.15;0.33) | 2.13x10^-07^ | 0.26 |
| 9 | chr9:22019057 | 22019057 | A (0.47) | 0.24 (0.15;0.33) | 2.09x10^-07^ | 0.30 |
| 9 | chr9:22019445 | 22019445 | A (0.47) | 0.24 (0.15;0.33) | 1.88x10^-07^ | 0.26 |
| 9 | chr9:22020027 | 22020027 | T (0.47) | 0.24 (0.15;0.33) | 3.65x10^-07^ | 0.22 |
| 9 | chr9:22021005* | 22021005 | G (0.44) | -0.23 (-0.32;-0.14) | 7.81x10^-07^ | 0.69 |
| 9 | chr9:22022119 | 22022119 | G (0.48) | 0.25 (0.16;0.34) | 1.32x10^-07^ | 0.20 |
| 9 | chr9:22026112* | 22026112 | G (0.43) | -0.23 (-0.32;-0.14) | 1.03x10^-06^ | 0.72 |
| 9 | chr9:22026367* | 22026367 | C (0.43) | -0.23 (-0.32;-0.14) | 7.11x10^-07^ | 0.72 |
| 9 | chr9:22030765* | 22030765 | C (0.43) | -0.23 (-0.32;-0.14) | 1.15x10^-06^ | 0.54 |
| 9 | chr9:22031443 | 22031443 | C (0.48) | 0.25 (0.15;0.34) | 1.41x10^-07^ | 0.19 |
| 9 | chr9:22033612 | 22033612 | T (0.37) | -0.19 (-0.29;-0.10) | 5.71x10^-05^ | 0.90 |
| 9 | chr9:22033819 | 22033819 | C (0.47) | 0.24 (0.15;0.33) | 3.92x10^-07^ | 0.17 |
| 9 | chr9:22038391 | 22038391 | T (0.47) | 0.24 (0.15;0.33) | 2.53x10^-07^ | 0.28 |
| 9 | chr9:22038414 | 22038414 | A (0.38) | 0.26 (0.17;0.35) | 4.38x10^-08^ | 0.83 |
| 9 | chr9:22038683 | 22038683 | A (0.47) | 0.24 (0.15;0.33) | 2.57x10^-07^ | 0.30 |
| 9 | chr9:22038859 | 22038859 | C (0.47) | 0.24 (0.15;0.33) | 3.10x10^-07^ | 0.32 |
| 9 | chr9:22039130 | 22039130 | G (0.47) | 0.23 (0.14;0.32) | 3.95x10^-07^ | 0.39 |
| 9 | chr9:22039656 | 22039656 | A (0.48) | 0.24 (0.15;0.33) | 3.15x10^-07^ | 0.23 |
| 9 | chr9:22040613 | 22040613 | G (0.48) | 0.25 (0.15;0.34) | 1.32x10^-07^ | 0.28 |
| 9 | chr9:22041670* | 22041670 | G (0.43) | -0.24 (-0.33;-0.14) | 4.76x10^-07^ | 0.47 |
| 9 | chr9:22042734 | 22042734 | T (0.43) | -0.23 (-0.32;-0.14) | 7.33x10^-07^ | 0.56 |
| 9 | chr9:22042810 | 22042810 | C (0.48) | 0.24 (0.15;0.33) | 1.61x10^-07^ | 0.34 |
| 9 | chr9:22043709 | 22043709 | G (0.48) | 0.24 (0.15;0.33) | 2.81x10^-07^ | 0.33 |
| 9 | chr9:22045048 | 22045048 | G (0.48) | 0.24 (0.15;0.34) | 1.66x10^-07^ | 0.21 |
| 9 | chr9:22046359 | 22046359 | A (0.44) | -0.23 (-0.33;-0.14) | 5.45x10^-07^ | 0.32 |
| 9 | chr9:22046499* | 22046499 | A (0.48) | 0.26 (0.17;0.35) | 2.35x10^-08^ | 0.11 |
| 9 | chr9:22050935 | 22050935 | A (0.41) | -0.26 (0.35;-0.17) | 4.34x10^-08^ | 0.43 |
| 9 | chr9:22051562* | 22051562 | C (0.42) | -0.26 (-0.35;-0.17) | 4.24x10^-08^ | 0.69 |
| 9 | chr9:22055657* | 22055657 | G (0.42) | -0.25 (0.35;0.16) | 6.25x10^-08^ | 0.50 |
| 9 | chr9:22057276* | 22057276 | T (0.42) | -0.25 (-0.35;-0.16) | 6.65x10^-08^ | 0.48 |
| 9 | chr9:22057830* | 22057830 | G (0.42) | -0.26 (-0.35;-0.16) | 4.88x10^-08^ | 0.52 |
| 9 | chr9:22058652* | 22058652 | G (0.42) | -0.26 (-0.35;-0.16) | 4.88x10^-08^ | 0.52 |
| 9 | chr9:22061750 | 22061750 | C (0.42) | -0.24 (-0.33;-0.15) | 2.85x10^-07^ | 0.63 |
| 9 | rs1063192* | 21993367 | G (0.44) | -0.20 (-0.29;-0.11) | 1.83x10^-05^ | 0.65 |
| 9 | rs1412829 | 22033926 | G (0.42) | -0.22 (-0.31;-0.13) | 2.91x10^-06^ | 0.41 |
| 9 | rs1537378* | 22051614 | A (0.42) | -0.25 (-0.35;-0.16) | 5.95x10^-08^ | 0.48 |
| 9 | rs2157719 | 22023366 | C (0.43) | -0.23 (-0.32;-0.14) | 7.88x10^-07^ | 0.69 |
| 9 | rs3217992* | 21993223 | T (0.38) | 0.24 (0.14;0.33) | 7.88x10^-07^ | 0.64 |
| 9 | rs523096* | 22009129 | G (0.45) | -0.21 (-0.30;-0.12) | 5.64x10^-06^ | 0.45 |
| 9 | rs543830* | 22016639 | T (0.42) | -0.22 (-0.31;-0.13) | 2.86x10^-06^ | 0.90 |
| 9 | rs564398* | 22019547 | C (0.42) | -0.22 (-0.31;-0.13) | 2.26x10^-06^ | 0.58 |
| 9 | rs615552* | 22016077 | C (0.44) | -0.21 (-0.30;-0.12) | 6.22x10^-06^ | 0.57 |
| 9 | rs634537* | 22022152 | G (0.42) | -0.23 (-0.32;-0.14) | 1.16x10^-06^ | 0.65 |
| 9 | rs679038* | 22019080 | A (0.42) | -0.22 (-0.31;-0.13) | 2.15x10^-06^ | 0.56 |
| 9 | rs7030641* | 22044040 | C (0.43) | -0.23 (-0.32;-0.14) | 1.03x10^-06^ | 0.63 |
| 9 | rs8181050* | 22054391 | G (0.42) | -0.25 (-0.35;-0.16) | 5.69x10^-08^ | 0.60 |
|  |  |  |  |  |  |  |

CHR: Chromosome number; SNP: single nucleotide polymorphism (some of the SNPs are represented as CHR: physical position); MAF: minor allele frequency from our data; CI: confidence interval; HWE: Hardy Weinberg equilibrium. No SNP was found in the clump belonging to rs9349379. *: SNPs which are identical in our study and the CHARGE CAC genome-wide association study.
