# Supplementary material for: Risk loci for coronary artery calcification replicated at 9p21 and 6q24 in the Heinz Nixdorf Recall Study
Source: BMC Med Genet. 2013 Feb 8;14:23. doi: 10.1186/1471-2350-14-23 (PMC3583714; doi:10.1186/1471-2350-14-23)
Supplement: Additional file 2: Table S2 — Result of the SNPs belonging to the same clump of SNPs associated with P ≤ 1x10^-05^ for loge(CAC score + 1). SNPs marked in bold are the top associated SNP of each clump. [file 1471-2350-14-23-S2.doc]

**Supplementary Table II:** Result of the SNPs belonging to the same clump of SNPs associated with P**≤** 1x10^-05^ for log_e_(CAC score+1). SNPs marked in bold are the top associated SNP of each clump.

| CHR | SNP | | Physical position | Minor allele (MAF) | BETA (95% CI) | P | HWE |
| --- | --- | --- | --- | --- | --- | --- | --- |
| **9** | **rs545226** | | **22002422** | **G (0.41)** | **0.23 (0.13;0.32)** | **1.28x10^-6^** | **0.31** |
| **6** | **rs10807323*** | **12903017** | | **A (0.41)** | **0.21 (0.12;0.30)** | **5.34 x10^-6^** | **0.73** |
| 6 | chr6:12909953 | 12909953 | | C (0.41) | 0.21 (0.12;0.30) | 7.60x10^-06^ | 0.74 |
| 6 | chr6:12933860* | 12933860 | | T (0.43) | 0.19 (0.10;0.28) | 2.84x10^-05^ | 0.43 |
| **4** | **rs4862857** | **189254052** | | **G (0.15)** | **0.29 (0.17;0.42)** | **6.24x10^-6^** | **0.15** |
| **5** | **rs303352** | | **15419156** | **C (0.33)** | **0.21 (0.12;0.31)** | **1.60x10^-05^** | **0.24** |
| **13** | **rs9546628** | | **83660874** | **G (0.06)** | **0.43 (0.23;0.63)** | **2.46x10^-05^** | **0.10** |
| **1** | **rs11587701** | | **13819084** | **G (0.07)** | **-0.39 (-0.57;-0.21)** | **2.67x10^-05^** | **0.64** |
| **22** | **rs12483930** | | **31899454** | **G (0.04)** | **0.51 (0.27;0.75)** | **2.87x10^-05^** | **0.69** |
| **22** | **rs2267260** | | **32407196** | **C (0.42)** | **-0.20 (-0.28;-0.11)** | **3.72x10^-05^** | **0.74** |
| **21** | **rs2832335** | | **29753004** | **T (0.11)** | **-0.30 (-0.44;-0.16)** | **3.86x10^-05^** | **0.21** |
| 21 | rs8132309 | | 29767744 | T (0.11) | -0.29 (-0.44;-0.15) | 6.31 x10^-05^ | 0.08 |
| **4** | **rs4619848** | | **30436903** | **T (0.48)** | **-0.19 (-0.28;-0.10)** | **4.25x10^-05^** | **0.46** |
| **3** | **rs12493885** | | **155322556** | **G (0. 13)** | **-0.28 (-0.42;-0.15)** | **4.29x10^-05^** | **0.84** |
| **4** | **rs7662582** | | **91988405** | **A (0.09)** | **0.32 (0.17;0.47)** | **4.70x10^-05^** | **0.13** |
| 4 | rs17248550 | | 91985634 | A (0.09) | 0.33 (0.17;0.48) | 2.85 x10^-05^ | 0.19 |
| **9** | **chr9:22091702** | | **22091702** | **T (0.31)** | **-0.20 (-0.30;-0.11)** | **5.02x10^-05^** | **0.58** |
| 9 | chr9:22018406 | | 22018406 | A (0.32) | -0.20 (-0.29;-0.10) | 7.45 x10^-05^ | 0.28 |
| 9 | chr9:22040898 | | 22040898 | A (0.33) | -0.20 (-0.30;-0.10) | 5.10 x10^-05^ | 0.19 |
| 9 | chr9:22054465 | | 22054465 | A (0.33) | -0.19 (-0.29;-0.10) | 9.98 x10^-05^ | 0.07 |
| 9 | rs1333040 | | 22073404 | C (0.44) | -0.22 (-0.31;-0.12) | 3.59 x10^-06^ | 1 |
| 9 | chr9:22077473 | | 22077473 | T (0.32) | -0.20 (-0.29;-0.10) | 9.68 x10^-05^ | 0.56 |
| **16** | **chr16:66485198** | | **66485198** | **T (0.03)** | **0.60 (0.31;0.89)** | **5.06x10^-05^** | **1** |
| 16 | chr16:66448575 | | 66448575 | C (0.03) | 0.59 (0.31;0.88) | 5.79x10^-05^ | 1 |
| **6** | **chr6:13061998** | | **13061998** | **A (0.15)** | **-0.26 (-0.39;-0.13)** | **6.02x10^-05^** | **0.11** |
| 6 | chr6:13059564 | | 13059564 | C (0.16) | -0.26 (-0.38;-0.13) | 4.35x10^-05^ | 0.12 |
| 6 | rs9473086 | | 13065021 | T (0.15) | -0.26 (-0.39;-0.13) | 6.94 x10^-05^ | 0.11 |
| 6 | chr6:13065398 | | 13065398 | T (0.15) | -0.27 (-0.39;-0.14) | 4.77 x10^-05^ | 0.11 |
| 6 | chr6:13065607 | | 13065607 | A (0.15) | -0.27 (-0.40;-0.15) | 3.98 x10^-05^ | 0.11 |
| 6 | chr6:13065811 | | 13065811 | G (0.16) | -0.25 (-0.37;-0.13) | 7.24 x10^-05^ | 0.15 |
| 6 | chr6:13067801 | | 13067801 | C (0.16) | -0.25 (-0.37;-0.12) | 9.71 x10^-05^ | 0.12 |
| 6 | chr6:13069206 | | 13069206 | G (0.16) | -0.25 (-0.37;-0.12) | 9.9 x10^-05^ | 0.15 |
| 6 | chr6:13090409 | | 13090409 | C (0.16) | -0.25 (-0.38;-0.13) | 6.51 x10^-05^ | 0.12 |
| 6 | chr6:13106875 | | 13106875 | T (0.18) | -0.24 (-0.36;-0.12) | 8.41 x10^-05^ | 0.11 |
| 6 | chr6:13125902 | | 13125902 | G (0.18) | -0.23 (-0.35;-0.12) | 8.57 x10^-05^ | 0.52 |
| 6 | chr6:13128303 | | 13128303 | A (0.18) | -0.24 (-0.36;-0.12) | 5.98 x10^-05^ | 0.19 |
| 6 | chr6:13131774 | | 13131774 | T (0.19) | -0.25 (-0.36;-0.13) | 2.76 x10^-05^ | 0.16 |
| 6 | chr6:13132522 | | 13132522 | G (0.19) | -0.25 (-0.36;-0.13) | 3.23 x10^-05^ | 0.19 |
| 6 | chr6:13132592 | | 13132592 | C (0.19) | -0.25 (-0.36;-0.13) | 2.76 x10^-05^ | 0.16 |
| 6 | chr6:13133550 | | 13133550 | T (0.19) | -0.25 (-0.36;-0.13) | 3.06 x10^-05^ | 0.17 |
| 6 | chr6:13135775 | | 13135775 | C (0.19) | -0.23 (-0.35;-0.12) | 7.25 x10^-05^ | 0.16 |
| 6 | chr6:13138979 | | 13138979 | C (0.20) | -0.23 (-0.35;-0.12) | 5.30 x10^-05^ | 0.33 |
| 6 | chr6:13139726 | | 13139726 | A (0.19) | -0.23 (-0.35;-0.12) | 7.79 x10^-05^ | 0.27 |
| 6 | chr6:13146865 | | 13146865 | G (0.19) | -0.25 (-0.36;-0.13) | 3.15 x10^-05^ | 0.23 |
| 6 | chr6:13160679 | | 13160679 | T (0.19) | -0.26 (-0.37;-0.14) | 1.4 x10^-05^ | 0.21 |
| 6 | chr6:13176945 | | 13176945 | A (0.22) | -0.23 (-0.34;-0.12) | 3.96 x10^-05^ | 0.76 |
| 6 | chr6:13194636 | | 13194636 | A (0.22) | -0.22 (-0.33;-0.11) | 9.9 x10^-05^ | 0.97 |
| **9** | **chr9:21992477** | | **21992477** | **A (0.33)** | **-0.19 (-0.29;-0.10)** | **7.78 x10^-05^** | **0.42** |
| **11** | **rs949126** | | **110815769** | **A (0.16)** | **0.24 (0.12;0.37)** | **8.74x10^-05^** | **0.25** |
| **6** | **chr6:13208807** | | **13208807** | **A (0.22)** | **-0.22 (-0.34;-0.11)** | **9.14 x10^-05^** | **0.93** |
| 6 | chr6:13212567 | | 13212567 | T (0.22) | -0.22 (-0.34;-0.11) | 8.59 x10^-05^ | 0.89 |
| **14** | **rs17726882** | | **45442585** | **C (0.15)** | **-0.25 (-0.38;-0.13)** | **9.69x10^-05^** | **0.95** |
| 14 | rs10138440 | | 45601722 | T (0.16) | -0.28 (-0.40;-0.16) | 8.92 x10^-06^ | 0.70 |
| **11** | **rs604904** | | **67040467** | **G (0.04)** | **0.44 (0.22;0.66)** | **9.73x10^-05^** | **0.36** |
| 11 | rs672432 | | 67033045 | T (0.04) | 0.47 (0.24;0.70) | 6.06 x10^-05^ | 0.24 |

CHR: Chromosome number; SNP: single nucleotide polymorphism (some of the SNPs are represented as CHR: physical position); MAF: minor allele frequency from our data; CI: confidence interval; HWE: Hardy Weinberg equilibrium. *: SNPs which are identical in our study and the CHARGE CAC genome-wide association study.
