# Supplementary material for: Risk loci for coronary artery calcification replicated at 9p21 and 6q24 in the Heinz Nixdorf Recall Study
Source: BMC Med Genet. 2013 Feb 8;14:23. doi: 10.1186/1471-2350-14-23 (PMC3583714; doi:10.1186/1471-2350-14-23)
Supplement: Additional file 3: Table S3 — Result of association for loge(CAC score + 1) with SNPs associated with coronary artery disease in CARDIOGRAM and coronary artery calcification in CHARGE. [file 1471-2350-14-23-S3.doc]

**Supplementary Table III:** Result of association for log_e_(CAC score+1) with SNPs associated with coronary artery disease in CARDIOGRAM and coronary artery calcification in CHARGE.

| CHR | Gene(s) | SNP | Physical position | Minor allele | BETA (95% CI) | P | HWE |
| --- | --- | --- | --- | --- | --- | --- | --- |
| 1 | *PCSK9* | rs11206510 | 55268627 | C (0.19) | -0.11 (-0.23;-0.005) | 0.06 | 1 |
| 1 | *SORT1* | rs599839 | 109623689 | G (0.23) | -0.13 (-0.24;-0.03) | 0.02 | 0.60 |
| 1 | *MIA3* | rs17465637 | 220890152 | A (0.26) | -0.12 (-0.22;-0.02) | 0.02 | 0.88 |
| 2 | *WDR12* | rs6725887 | 203454130 | C (0.13) | -0.02 (-0.16;0.11) | 0.75 | 0.59 |
| 3 | *MRAS* | rs2306374 | 139602642 | C (0.18) | 0.26 (0.13;0.38) | 2.7x10^-05^ | 0.75 |
| 6 | *PHACTR1* | rs12526453 | 13035530 | G (0.35) | -0.17 (-0.27;-0.08) | 2.9x10^-04^ | 0.25 |
| 6 | *LPA* | rs3798220 | 160881127 | NA | NA | NA | NA |
| 9 | *CDKN2A/2B* | rs4977574 | 22088574 | NA | NA | NA | NA |
| 10 | *CXCL12* | rs1746048 | 44095830 | T (0.14) | -0.19 (-0.32;-0.05) | 0.006 | 0.75 |
| 12 | *SH2B3* | rs3184504 | 110368991 | C (0.49) | -0.01 (-0.10;0.08) | 0.81 | 0.49 |
| 19 | *LDLR* | rs1122608 | 11024601 | T (0.25) | -0.10 (-0.20;0.01) | 0.07 | 1 |
| 21 | *MRPS6* | rs9982601 | 34520998 | T (0.14) | 0.15 (0.02;0.28) | 0.02 | 0.26 |
| 1 | *PPAP2B* | rs17114036 | 56735409 | G (0.09) | -0.18 (-0.35;-0.01) | 0.04 | 0.26 |
| 6 | *ANKS1A* | rs17609940 | 35142778 | NA | NA | NA | NA |
| 6 | *TCF21* | rs12190287 | 134256218 | G (0.36) | 0.03 (-0.06;0.13) | 0.48 | 0.52 |
| 7 | *ZC3HC1* | rs11556924 | 129450732 | T (0.39) | -0.05 (-0.15;0.04) | 0.28 | 0.73 |
| 9 | *ABO* | rs579459 | 135143989 | C (0.24) | 0.13 (0.02;0.23) | 0.02 | 0.51 |
| 10 | *CNNM2, NT5C2* | rs12413409 | 104709086 | A (0.10) | 0.09 (-0.06;0.24) | 0.26 | 0.46 |
| 11 | *APOA5-A4-C3-A1* | rs964184 | 116154127 | NA | NA | NA | NA |
| 13 | *COL4A1/A2* | rs4773144* | 109758713 | G (0.44) | 0.06 (-0.03;0.15) | 0.19 | 0.17 |
| 14 | *HHIPL1* | rs2895811 | 99203695 | C (0.43) | 0.04 (-0.05;0.13) | 0.41 | 0.16 |
| 15 | *ADAMTS7* | rs3825807 | 76876166 | G (0.45) | -0.15 (-0.24;-0.06) | 0.001 | 0.20 |
| 17 | *SMG6, SRR* | rs216172 | 2073254 | NA | NA | NA | NA |
| 17 | *RASD1, SMCR3, PEMT* | rs12936587 | 17484447 | A (0.45) | -0.03 (-0.12;0.06) | 0.5 | 1 |
| 17 | *UBE2Z, GIP, ATP5G1* | rs46522 | 44343596 | NA | NA | NA | NA |
| 13 | *COL4A2* | rs3809346* | 109758944 | A (0.43) | 0.06 (-0.035;0.15) | 0.23 | 0.18 |

CHR: Chromosome number; SNP: single nucleotide polymorphism (some of the SNPs are represented as CHR: physical position); MAF: minor allele frequency from our data; CI: confidence interval; HWE: Hardy Weinberg equilibrium. NA: not available in our study. *: SNPs which are identical in our study and the CHARGE CAC genome-wide association study
